# Promoting Business and Entrepreneurial Awareness in Health Care Professionals: Lessons From Venture Capital Panels at Medicine 2.0 Conferences

**DOI:** 10.2196/jmir.3390

**Published:** 2014-08-06

**Authors:** Talya Miron-Shatz, Itamar Shatz, Stefan Becker, Jigar Patel, Gunther Eysenbach

**Affiliations:** ^1^Center for Medical Decidion MakingOno Academic CollegeKiryat OnoIsrael; ^2^Tel Aviv UniversityTel AvivIsrael; ^3^Department of NephrologyUniversity Duisburg-EssenEssenGermany; ^4^Institute for Drug SafetyUniversity Hospital EssenEssenGermany; ^5^London OfficeMcKinsey CompanyLondonUnited Kingdom; ^6^JMIR PublicationsToronto, ONCanada; ^7^University Health Network, Techna Institute, and University of TorontoToronto, ONCanada

**Keywords:** start-up, entrepreneurship, health technology, capital funding, telehealth, eHealth, mobile health, health technology, technology transfer, health 2.0, business pitch, entrepreneurship programs

## Abstract

There are few mechanisms that bring the academic and business worlds together in a way that would maximize the success of health technology (health tech) start-ups by increasing researchers’ knowledge about how to operate in the business world. Existing solutions (eg, technology transfer offices and dual degree MD/MBA programs) are often unavailable to researchers from outside the institution or to those who have already completed their primary education, such as practicing physicians. This paper explores current solutions and offers a partial solution: include venture capital (VC) panels in medical conferences. These VC panels educate academics on 2 important and interconnected issues: how to “pitch” their ideas in the business world and what to consider when creating a company. In these sessions, academia-based start-up companies present their ideas before a VC panel composed of professional investors and receive feedback on their idea, business plan, and presentation techniques. Recent panel recommendations from Medicine 2.0 conferences fell into 7 categories: (1) the product, service, or idea you are developing into a company, (2) determine market forces and identify the target audience, (3) describe your competitive advantage, (4) the business plan, (5) current and future resources and capabilities, (6) legal aspects, and (7) general advice on the art of pitching. The academic and business literature validates many of these recommendations suggesting that VC panels may be a viable and cost-effective introduction to business and entrepreneurial education for physicians and other health care professionals. Panels benefit not only the presenting companies, but also the physicians, psychologists, and other health care professionals attending the session. Incorporating VC panels into academic conferences might also illuminate the need for incorporating relevant business training within academia.

## Introduction

There is currently a disconnect between academia and business: researchers lack significant business training during their education. This gap in training, which stems from the focus on professional education (eg, in medicine), can impair academic researchers’ potential integration into the world of entrepreneurship and business management because they lack the business training to know i how to build a healthy business model [1]. This lack of business training for health care professionals is neither new nor unknown. A study from 1993, for example, demonstrated that only 3% of young physicians (younger than 45 years) felt that they were well prepared to manage the business aspects of medical practice [2]. Today, psychologists, physicians, and other academics are increasingly developing interventions for health improvement and disease prevention, yet the leap into large-scale implementation of these interventions usually requires business knowledge. Without this knowledge, researchers are often unable to successfully develop their ideas commercially and they cannot manage to turn them into successful products or companies [3].

Our overwhelming sense that the field is alive with effective interventions that do not later translate to scalable products or services impelled the creation of the venture capital (VC) panel at the Medicine 2.0 conferences. The disconnect between academia and business (or “industry”) can be demonstrated in 2 noticeable areas: the curricula in medical schools and the agendas of academic conferences. These 2 areas offer barriers, but also hidden opportunities; by modifying them to include the discussion of the integration between science and business, we can bridge the current gap and increase researchers’ knowledge about how to operate in the business world. In this paper, we seek to cast light on the academia-business gap, illuminate existing solutions and limitations, and offer a partial remedy that provides business education in a nutshell. Additionally, we hope that this solution will make health care professionals realize what is missing in their training, and therefore will stimulate demand for changes within the medical curriculum and training process.

To highlight the knowledge gap, we first examined the curriculum of Harvard Medical School. Recently ranked as the number 1 medical school for research in the United States [4], Harvard Medical School does not currently require its graduates to take any business classes as part of their education [5]. Thus, even after completing the 4-year program, most certified physicians who graduate from Harvard Medical School have little to no formal business training. This may hamper their professional development when they begin actively leading research in the medical field, running private practices, or creating health-related start-ups (if they decide to do this) [6].

Second, we examined the agendas of several prominent academic conferences and found that they focused solely on science, ignoring business implementation altogether. Some notable examples are the International Federation of Fertility Societies and the American Society for Reproductive Medicine Joint Annual Meeting [7], the British Academy of Audiology Annual Conference [8], and the World Congress of the World Society for Pediatric Infectious Diseases [9]. None of these conferences offered any business-related sessions during their 2013 events. Interestingly, even one of the biggest international medical trade fairs, Medica in Dusseldorf, Germany, has not staged a VC panel session thus far [10]. Even conferences that are attuned to the issue of implementation, such as the NHS Health and Care Innovation Expo, showcase innovations helping visitors to bring about changes, improvements, and renewals within the NHS benefiting the whole community, but do not include a panel to inform medical entrepreneurs on how to bring their solutions to the stage where they can benefit the entire community [11]. A welcome exception is the Doctors 2.0 & You conference [12], which concentrates on understanding how physicians use new technologies, such as Web 2.0 and social media, and the impact of these latest technologies on the relation between physicians and patients, colleagues, industry, and the public sector. Perhaps because of its focus on integration with the industry, the first session is a start-up contest bringing together 7 companies from 5 countries working on diverse aspects of digital health. Attendees are expected from a range of industries, including the public, and physicians, professional and patient associations, pharmaceutical companies, governments, and insurance companies [12].

Interestingly, the American Medical Association (AMA) Accelerating Change in Medical Education Conference (held in Chicago on October 4 and 5, 2013) brought together almost 200 leaders in medical education from across the United States to discuss innovations needed to bridge the gap between the training of medical students and the needs of the health care system. Although there was an opportunity to learn about the grant projects supported by the Accelerating Change in Medical Education initiative, there was no VC panel [13]. Because conferences can instruct and set an agenda for a field, this is a missed opportunity.

The lack of business training has adverse effects on doctors and other health care professionals’ forays into the world outside of medical school. A recent article featured on the Cancer Network website [14] showed that many doctors are unaware of the significance of having a proper business plan for their practice and are often unable to design one even if they do grasp its importance. Therefore, many struggle financially while running their practices. The detrimental effect of the lack of business education is exacerbated when it comes to more complex financial and business issues. Specifically, the chief executive officer (CEO) of a patient relationship management company [15] lists the most common reasons for the failure of health technology (health tech) start-ups: a lack of specific focus or adoption point, misunderstanding the consumers’ willingness to pay for the service or how much effort they would be willing to expend to use it, requiring too much money for development of the product, having too complex an organizational structure, and lacking understanding of reimbursement dynamics. The same problems were raised in an article explaining why business modeling is crucial in the development of eHealth technologies [16] and in an article that discussed the importance of understanding business and economic strategies during the development of eHealth solutions [17].

Furthermore, a lack of understanding of business models reduces the ability of start-ups conceived in academia to receive funding for their development. The present paper focuses on fundraising from private sources (primarily from VC funds as discussed subsequently). However, the need to present a convincing case for the viability of an idea from a business perspective also applies when seeking to raise money from government sources such as federal grants [18], an important source of funding for health care start-ups [19]. Each source of funding has its own merits and is aimed at people and companies with different goals, although considerable overlap does exist between the 2 sources. Some examples are the emphasis on assembling a skilled team, showing the need for the proposed solution, and explaining why it should work [20-21]. In both cases, compelling arguments assist in securing funding. The primary difference is that federal grants are generally aimed at scientists who require additional funding to further their academic research in congruence with their university [22], whereas VC funding is aimed at companies looking to expand and explore commercial opportunities for profit [23]. Thus, the latter places more emphasis on larger growth or commercialization independent of a host academic institution.

All these issues share a single commonality: scientists lack a proper introduction into the intricacies of the business world and, therefore, risk being in a suboptimal position to develop their idea into a working marketable concept.

## Existing Solutions for Bridging the Academic-Industry Gap

There are 4 main solutions currently in place that aim to minimize the adverse effects of the problem. These are technology transfer offices, entrepreneurship centers, specialized entrepreneurship programs, and medicine/business dual degree programs. However, none of these solutions will solve the problem entirely.

The first solution is the technology transfer offices (tech transfer) present in many universities, companies, and government organizations [24]. Their role is to identify which research has potential commercial interest and how to best develop and use it [25]. Although they serve an important purpose, many tech transfers do not comprehensively educate scientists about how the business world works [26-28]. Although they have a definite positive impact on research development, tech transfers are an incomplete solution because, in our opinion, many fail to give researchers the tools necessary for them to flourish and succeed in navigating the business aspects of the health care industry.

The second solution that sets out to deal with scientists’ lack of business experience is the establishment of entrepreneurship centers in universities. These centers provide valuable support and training to aspiring entrepreneurs or researchers who are interested in learning more about the business world [29]. Unfortunately, although these centers provide obvious benefits, their greatest drawback lies in their locality because they are inherently limited in their ability to help anyone outside of the specific university in which they are set up. For example, the Global Consortium of Entrepreneurship Centers (GCEC), which is the premier organization to promote cooperation between entrepreneurship centers from different universities, is currently comprised of over 200 centers across the United States [30]. However, this is limited in scope because researchers from universities without these entrepreneurial centers rarely benefit from this sort of support.

The third available solution is specialized entrepreneurship programs that provide business education to scientists, such as the Stanford Summer Program on Bio-Entrepreneurship [31]. These entrepreneurship education and training (EET) programs teach scientists how to develop their research into a viable product or a functioning company. A quantitative review of all literature on the subject showed that EETs have a positive impact on entrepreneurial success [32]. The study found a statistically significant relationship between EET and entrepreneurship-related human capital assets (*r*=.217) and between EET and entrepreneurship outcomes (*r*=.159). More importantly, the study showed that the relationship between EET and entrepreneurship outcomes is stronger for academic-focused EET interventions (*r*=.238) than for training-focused EET interventions (*r*=.151), which emphasizes the importance of EET for academics. Again, the shortcoming is that EETs are a localized solution with limited coverage. Despite having a definite positive impact, EET programs cannot reach most health care professionals and researchers.

The fourth solution is a combined Doctor of Medicine (MD) and Master of Business Administration (MBA) program. These dual degree programs are designed with the goal of training physicians who are skilled in both medicine and business management. The integrated curriculum is designed in a way that strives to increase the drive, enthusiasm, and ambition of the degree candidates, containing the most important concepts from both fields: from strategy, finance, marketing, and economics on the business end to anatomy, physiology, biochemistry, and all other related core science disciplines of medicine [33,34]. Such programs are currently available in over 50 universities around the United States [35]. Dual degree programs are also effective in that students who participate in a dual degree program often perform better academically and have a higher degree of satisfaction with their studies than students who complete only an MBA or Doctor of Pharmacy (PharmD) program [36,37]. Although they offer the best and most extensive form of combined training (as far as receiving a business and a medical education goes), these dual degree programs suffer from a shortcoming similar to the one mentioned previously: anyone who did not study in such a program is unable to benefit from their existence. In addition, there is a scarcity of similar programs accessible to physicians during or immediately after residency training [38]. Lately, distance learning and online technology have permeated all levels of business education. However, most profiled programs so far are for general MBAs rather than combined MD/MBAs [39]. None of the courses featured by the Financial Times’ Online MBA Listing 2014 focused on health care. Once again, the solution falls short because it reaches only a relatively small portion of the health care population.

For some start-ups, “incubators” may play an important role. These programs are designed to support researchers coming up with ideas by providing an array of business and services resources. Key to the success of such cooperations are powerful networks, in which all partners can trust. These are vital for bringing together know-how and venture capital. Yet, incubators are found outside of academic settings.

## A Partial Solution and Potential Catalyst for Change: Venture Capital Panels in Medical Conferences

Having established the existence of a problem—the lack of business training for health care professionals—and the drawbacks of current solutions, we would like to propose an additional (although partial) solution, which overcomes the locality issue, namely VC panels hosted in medical and health care conferences. We are aware that VC panels cannot solve the problem entirely. In fact, anything short of making extensive business classes mandatory in medical school is unlikely to be a perfect solution. However, we believe that VC panels are a highly time- and cost-effective means of getting exposure to a broader sample of health care professionals. For many attendees, this can be their first substantial interaction with the business aspects of the research world. Deciding to attend a 90-minute session is not as big a time or financial commitment as deciding to enroll in an MBA degree, for instance. Because of this, VC panel sessions in academic conferences may attract people who are only in the early stage of considering business training or are exploring the relevance of the business world to their practice. Thus, these sessions can serve as a catalyst for creating demand for business education to be included in medical and other training and continued education programs.

Venture capital is funding provided to start-up companies. A VC fund receives equity in the company in return for its investment [40]; therefore, they tend to be long-term investments [41]. VC investments generally occur after a seed-funding round (used to start the business) has already taken place, although some funds also invest at the seed stage [42]. In 2010, there were 462 active (investing at least US $5 million) VC firms in the United States who invested approximately $22 billion into nearly 2749 companies, 1001 of these companies receiving funding for the first time [43]. Business factors, such as the potential for rapid return on investment and a credible business plan, are generally considered more important than product characteristics [44].

A VC panel is where companies and start-ups present their idea to venture capitalists in front of an audience and they are often included in industry conferences, events, and television shows, such as *Shark Tank* or its UK equivalent *Dragon’s Den*. A number of prominent events developed in the United States over the past 7 years within the field of health information technology (IT). Examples are the Venture+ forum at the Health Information Management Systems Society (HIMSS) conference (Venture+ 2014: Health IT and Partnering Forums [45]), the Telemedicine Venture Summit at the conference of the American Telemedicine Association (American Telemedicine Association 2014 [46]), and the HealthTech Conference [47]. All combine educational components with possibilities for start-ups (between 10 and 45 companies) to present themselves to a panel. In 2013 and 2014, most topics revolved around mobile health (mHealth), in particular, patient-doctor communication. Benchmarking of the events is difficult because the number of applicants, growth attendance, and criteria for selection is not always made publicly available. One example comes from the HealthTech 2013 Conference, which hosted the “Grand Rounds Innovation Showdown.” During this event, 10 start-up companies in the health industry (chosen out of more than 150 applicants) pitched their product or service to a group of judges, in front of a crowd of more than 400 health care executives, IT decision makers, venture capitalists, and members of the press [47]. Unfortunately, no reliable data exist in examining the extent to which VC panels have affected the development of companies in which they have invested. Similar events in Europe are relatively rare: The Charité Entrepreneurship Summit has only recently started focusing on IT (Charité Entrepreneurship Summit 2014 [48]). The biggest Medical IT conference, Connecting Healthcare IT (conhIT), has not offered VC panels thus far [49].

The benefits of including VC panels in academic conferences extend both to the companies presenting and to the audience. The companies receive invaluable feedback and get to practice “pitching,” an essential skill in the business world [50] that is not a part of the academic training process. For the audience, the benefits include hearing about innovative new companies, learning from the feedback the companies receive, and becoming more familiar with pitches and company presentations. Panel members also benefit from an early glimpse at cutting-edge scientific developments and from exposure to existing and future academic entrepreneurs. Networking opportunities abound for all parties involved.

## Venture Capital Panels at the 2012 and 2013 Medicine 2.0 Conferences

The Medicine 2.0 conference, established by Gunther Eysenbach in 2008, focuses on subjects such as digital disease detection, health information on the Web, and business models in a Web 2.0 environment [51]. This conference is perfectly positioned for beginning to bridge the gap between industry and academia, and for suggesting a new agenda. It showcases studies by researchers who either developed interventions for improving health and the transfer of health information, or are evaluating existing practices. In an era of burgeoning innovation and technological advancement in health care, there is great opportunity to marry the 2 sides. We propose to achieve this not only by introducing academics to investors, but also from providing academics with the knowledge and know-how of turning their validated ideas into businesses.

For the past 2 years (2012 and 2013), the Medicine 2.0 conference included a start-up panel organized and chaired by Professor Talya Miron-Shatz, a decision scientist, industry consultant, and CEO of CureMyWay, a behavior change start-up. During the panel sessions, companies conceived inside or alongside academic institutions presented their ideas to investors and other stakeholders, and received feedback that also served to inform the audience in attendance of the requirements of obtaining funding from such sources.

Members of the panels in 2012 and 2013 were seasoned investors: William Cowen of Long River Ventures, Boston; Joseph Kvedar of Health Partners, Boston; Jay Mohr of Locust Walk Partners, Boston; Jigar Patel of McKinsey & Company, London; Sid Thekkepat of m8capital, London; and Jack Young of Qualcomm Ventures, San Diego.

The companies that presented to the 2012 and 2013 VC panels had interesting and novel ideas in various stages of development. They ran the gamut from a person with an idea, 2 people developing a service, a company that had already established an impressive advisory board and raised funds, and many variations in-between. Their ideas included query engines for medical information, an online teenager community for maintaining a healthy body image, a system incorporating cell phone cameras with real-life Petri dishes to test water quality in Africa and elsewhere, a platform facilitating medical research, a health app, and a system for providing physicians with the most-read articles in their field. This suggests that Health 2.0 entrepreneurs can found companies based on a wide range of capabilities. The panel feedback reveals similarities in business needs, despite broad diversity in start-up topics.

For many researchers, the VC panel was an eye-opening first encounter with the business world. Therefore, we aggregated the feedback from the panels and compiled a list of the most critical pieces of information that the panelists related to companies. Entrepreneurs need to consider all the points mentioned subsequently when preparing a business presentation, but they are also crucial when developing the business idea and the company itself. In addition to this benefit, the feedback from the panels can help to outline and prioritize the subjects that entrepreneurial programs cover.

Table 1 lists the specific topics that companies were required to include in their pitch, with an example from a fictitious company. In this example, the fictitious company developed an apparatus for avoiding spillage when applying eye drops. Although the pitch was only 6-7 minutes long, presenting companies were required to cover all relevant topics.

The remainder of this paper outlines lessons learned from the VC panels, validates these lessons using current scientific and business literature, and discusses the potential implementation of VC panels as a partial yet scalable solution to health researchers’ lack of familiarity with the business world.

**Table 1:** Topics to be addressed in a business presentation (pitch) for a hypothetical product to reduce eye drop spillage.

Topic	Example
The need or the problem	Patients applying eye drops spill 30% of the drops outside their eye.
The current state of affairs	Unless someone helps the patient, there is 30% spillage. No gadgets exist to solve the problem.
The company’s solution	A mechanical device that is placed on the eye. The eyedrops bottle is placed in it. This ensures the bottle stays steady and there is less spillage.
Why the company’s solution is better than other solutions	It is cheap to produce and therefore affordable, it minimizes spillage by 70%, it can be sterilized, and it requires no special skill to use.
The market	100 million people worldwide apply eyedrops at least once a day.
Monetization	The device will be distributed by medical insurers to ensure efficacy of eyedrops and reduce medication waste, which leads to repurchase or the device will be sold to directly to consumers.
Development phase: technologically	There is a currently a fully functional prototype.
Development phase: team	There is an ophthalmologist on board as a chief scientific officer, an engineer as a CEO, and 2 graduate engineering students on the development team.
Funding so far	An NIH grant of $300,000 for 1 year, borrowed $45,000 from friends and family, and received a $100,000 angel investment.
Business proposition for investors: how much the company is looking to raise and under what terms	Seeking $1,000,000 for a postinvestment evaluation of $3,000,000.

## Venture Capital Panel Recommendations at the 2012 and 2013 Medicine 2.0 Conferences

The panelist comments (from 2012 and 2013) converged into 7 key areas, explained subsequently.

### The Product, Service, or Idea You Are Developing Into a Company

Similar to the introduction section in a scientific paper, as the presenter you need to assume that the people you are presenting to are intelligent, but not necessarily familiar with the specific issue or field you are working on. Again, like an introduction section, presentations require that you cover certain points before describing your results—or product in the case of VC panels.

1. Background: What problem does your product solve? Describe the current state of affairs, such as the magnitude of the problem. For example, “100 million people apply eye drops each day. Studies show that 25% of the active material is lost due to improper application. This reduces the effectiveness of the drops, causing drug switches, unnecessary doctor visits, and a 12% increase in eye infections.” Note that the background is based on scientific findings, but is very succinct and presented using simple terms. Information to include encompasses several aspects of the product, which go beyond the technical description of how the product operates. Specifically, in order to convince investors of the potential success of the product, the company needs to make educated prediction regarding usage and acceptance of the product, by consumers (patients), as well as other stakeholders, such as insurers and physicians.
  - What is your solution (the product)? This should be a concise description that people from outside of the industry will be able to understand.
  - How will the costumer use the product? This ties in with the description of the product and shows what sort of a relationship the target costumer will have with the product.
  - Are people willing to pay for your product? This dovetails with the questions regarding the business plan, subsequently, and should be backed up with facts (eg, market research, surveys, and similar product histories).
2. Stakeholder analysis: What are the issues that matter to people who might later wish to use the solution and to those who would be willing to pay for it? Particularly in the complex health care arena, consideration needs to be given to any group or individual who can affect the achievement of your company’s objectives or is affected by them [52]. For example, “Health insurers are paying for spilled medication. They want to increase efficiency of application to reduce the need for repeated purchases of the drops. Insurers want patients’ health to improve or be steady because the insurer pays for additional treatments required due to deterioration. Patients suffer discomfort from spillage and from reduced effectiveness of the eye drops.”

### Determine Market Forces and Identify the Target Audience

1. What market are you targeting and how big is it? This can be as specific as necessary to support the value of the product, but should be specific (eg, “Payers are spending US $20 million dollars in wasted eye drops each year”) rather than general (eg, “Health care in the United States is a US $3.8 trillion industry”).
2. Who are your competitors? In order to scan the competitive arena, you need to look beyond potential competitors and assess the competitive forces that can affect prospective profits [53]. A relevant question posed by panelists in this context is “What is your barrier to entry?” A *barrier to entry* is something that would stop your competition from developing a similar solution quickly and easily. A barrier could be an exclusive agreement you have already signed with major hospitals or health insurers, a patent, or anything else that requires ample time and/or money or other resources to develop, such as regulatory approval certification. Furthermore, you need to ask what degree your company is dependent upon suppliers and whether there are substitute offerings that could lure potential customers away.
3. Who is your customer? In the medical realm, customers can be divided into the 4 Ps: patient, provider, physician, and payer. There is also an important group that spans patient and provider that some products will directly target, namely caregivers. As a group, caregivers have a significant influence on the decisions individual patients make. In the previous example, the customer may be a pharmaceutical company that wants to differentiate its eye drops from others’ through using your device, a health insurer who wants to increase efficiency and reduce medication costs, or patients wishing to avoid the frustrating spillage. Likewise, an ophthalmologist can recommend the product to her patients to maximize efficiency and improve care.
4. What is your ability to ensure consumer engagement and loyalty? Engagement is increasingly becoming a parameter for evaluating companies that provide not just a service but also an experience to the user and should be quantified where possible.

### Describe Your Competitive Advantage

Pitching without describing your competition, even briefly, is like writing an academic paper without citing any literature. Showing that competitors exist does not mean there is not room for your company. Rather, this is a positive because it indicates that a market exists for your product.

1. What are your competitors’ approaches to the problem? Are they currently successful (growth rate, revenue, etc)? What does their success/failure mean for you?
2. How are you different from other services? This is sometimes also referred to as your differentiator: the feature or element that will make customers choose you over the competition.
3. How sustainable is your competitive advantage? How quickly could competitors imitate your strategy? How quickly may resources become unavailable?

### The Business Plan

The previous questions suggest that in order to pitch well, you need to be very familiar with the competition and to integrate these lessons into the building of your own product.

What is your revenue model (“show me the money”)? Including projected incomes and expenses, this is probably the biggest difference between the VC panel, which emphasizes financial sustainability, and the rest of the academic conference, which revolves around ideas, scientific findings, and implementation.

1. How are you going to make money or, in business jargon, to “monetize”? What is the payment model? If you plan to earn money primarily through reimbursement, does your model actually function? How long does it take to get paid?
2. Do you have an exit strategy? In other words, is there a feasible scenario for selling of your company or service that would no longer require your involvement? This is something VC investors seek because they expect a high return on their investment.
3. What is the lifetime value of a customer versus the cost of recruiting a customer? The bigger the gap between the 2, the more lucrative your business proposition. This relates to the question of how long they will a customer/patient use the product.
4. How much capital have you raised so far and how? This includes any personal financial stake that you have in the company. For example, “We have already raised US $50,000 from personal savings and angel investment.”
5. How much capital do you need and how are you planning to raise it? You should be able to justify the required capital and be able to explain what you plan to spend it on over a given time period (eg, staff costs, patenting your ideas, developing a prototype, or expanding the business to other markets). For example, “We are looking for US $1.2 million to fund an 18 month rollout of our product to the top 20 payer systems in New England by recruiting a product manager, sales force, marketing department, and investing in research and development to improve product quality and reduce manufacturing costs.”
6. What business proposition are you looking to offer investors: how much money and under what terms? How much of your company are you willing to give up in exchange for the funding? It is crucial to be aware of this and have a plan before approaching the negotiations table.

### Current and Future Resources and Capabilities

1. What phase is the company in technologically? How developed is the product? How far ahead are you in bureaucratic procedures such as patent filings and Food and Drug Administration (FDA) approvals?
2. What phase is the company in as far as a team is concerned? How experienced are team members? How well do they work together? Are they fully dedicated to the company (eg, what stake do they have in the company and what incentives do they receive)?
3. What are your monetary and development goals? What is the timeframe for the development of the business? Is it possible to accelerate progress using additional funding? Are there any potential bottlenecks that could hinder development? Are there any crucial deadlines?
4. Is your idea scalable and how? Scalability is the company’s ability to expand and deliver its products and services to multiple clients in various locations in a cost-efficient manner. In a digital world, this is simpler than it used to be. Scale, a prerequisite to growth, needs to be demonstrated.

### Legal Aspects

1. How are you dealing with intellectual property laws? This is particularly pertinent to companies that evolved in a university setting, where the intellectual property often belongs to the institution, not the researcher.
2. How are you dealing with privacy laws? Data ownership needs to be established, as does adherence to regulations such as those determined by HIPPA [54]. As shown by Miron-Shatz and Elwyn [55], most patients will not be aware of breeches to the privacy of their data, but such breeches occur consistently. For example, if a company offers a platform where physicians can share pictures of various ailments (even if patient information is deindividuated, so they cannot be identified), its founders need to ensure that this is in compliance with the Health Insurance Portability and Accountability Act (HIPAA) and other regulations because patients have ownership of their own pictures, meaning these may not be able to be shared by others without clear permissions in place.

### General Advice on the Art of Pitching

Apart from the content, the style and conveyance are also important, as business success hinges on impactful pitching. Similar to writing a scientific paper, having the data and the results is crucial, but the authors also need to present their arguments in a compelling manner so the journal accepts them (Textbox 1). Specific tips for presentation purposes were:

1. Be as focused and concise as possible. Both investors and your audience have a short attention span.
2. Use clear communication. Commercializing is a skill and has a language of its own. Beyond that, your communication needs to be clear and simple. Many comments revolved around the need to explain what the company does, from a number of angles, and in plain language. Make sure what you say is intelligible to people who are unfamiliar with the specific domain you operate in. On the first presentation slide, include a one-line description of your product/service (eg, “OpenTable for doctors”) so the panel and the audience will immediately know what your company does.
3. Use examples to highlight the need for your product and to show how you solve this need better, faster, and/or cheaper than anyone else does. You can do this by using cases of “the day in the life of...” a patient, physician, etc. This is the easiest way to show the panel how the product or idea works.
4. Show a lot of energy for your product to demonstrate that you believe in it and you will make it a success. This energy is something that a standard academic talk may lack because it is less of a “show” and more of a serious scientific presentation.

**Correlates of academic and business presentations.**

One of the 2013 panelists, Jigar Patel, who has a PhD in computer science and artificial intelligence, related how he prepared for his first academic talk, some years ago. He mentioned preparing ferociously for hard questions he thought he might be asked, but spending too little time thinking about the story he was about to tell, recapping his results in a compelling manner.

This anecdote demonstrates that pitching is an acquired skill, which everyone, including those currently well versed in business lingo, had to learn and master at some point. This is similar to the challenge a company faces when pitching its business idea to prospective investors or business partners.

In a way, a business presentation is not different from writing the abstract of a scientific paper, which needs to convince its reviewers that it is worth publishing and its potential audience that it is worth reading. Just like a scientific paper, business presentations also have their logic and acceptable structure.

### Final Words of Advice From the Panelists

1. Create barriers to entry by making it hard for others to imitate what you do. Accomplish this through a great user experience, intellectual property and patents, and/or through distribution channels and exclusive partnerships. The goal of this is primarily to protect yourself from intellectual theft of your product. This also makes it harder for other companies to compete with you directly by stealing your designs or methods.
2. Do not take it personally! Funders may choose not to invest just because they are in a late stage in the life of the fund, which means they are reserving money for continued investments in existing enterprises. There are many reasons why a funder may think your idea is brilliant, but still not invest.
3. Add value before seeking VC funding so you can retain more control in your company. Do so by looking for alternative sources of funding: collaborations, disease state groups, or nondilutive funding (eg, grants). These alternative sources of funding will likely also require a compelling business plan, pitch, or a proposal.
4. Practice makes perfect. Pitch to friends, colleagues, and mentors to get feedback before going to VCs. Consider filming yourself on video—this is a very honest way of realizing how you come across when you pitch. Get all the coaching and mentoring you can from people who will give you honest critical constructive feedback and give it your best shot.

## Validating Venture Capital Panel Feedback Against Contemporary Business Advice

When examining the academic literature on the subject, we found that our advice to presenting companies about how to make a successful pitch and how to create a business plan was similar to that included in published business books and articles [56-61]. Business authors recommend defining the target market, identifying revenue mechanisms, and considering the competitive strategy. In addition, the strength of arguments is dependent on the passion, enthusiasm, credibility, interpersonal behavior, social signals, and honesty driving a fact-based presentation [60-64]. A number of articles from popular business magazines offering “golden rules” or “typical mistakes” resemble the advice we gave participants at the Medicine 2.0 VC Panel [65-69]. Among typical mistakes were not being concise during the pitch (eg, “the elevator pitch is longer than 1 minute” or “the PowerPoint presentation is too long”), not having a factually supported, well-written executive summary (which is a less-detailed version of a business plan), overlooking a realistic exit strategy for investors, and taking things personally (“failure to listen”) [67]. The “10 Tips Successful Business Pitch Presentation” on the Harvard Entrepreneurships website complements our panels’ conclusions [68]: “find the right investors to pitch to” and “let the investors ask themselves why they should join you.” Research findings that supplement our experience comes from a study that coded 11 episodes of the Dutch *Dragons’ Den* television show. During these episodes, 43 people pitched their new products to 5 investors. The author found that whether the language of the pitch was concrete or abstract did not impact investment decisions. However, pitchers who had more knowledge than what was included in the pitch, such as the market, target audience, and patents, had 6 times greater chance of receiving an investment [70]. It also verifies the need for a succinct presentation: “An investor pitch is a comprehensive plan that can be communicated according to the “rule of 3.” There are moments where you have to communicate your plan in less than 3 minutes.” [70]. This is especially relevant during the early stages of the pitching process. During the later stages of negotiations, times allow for 30 minutes or even 3 hours of presentation and discussion [70]. We can confidently conclude that the feedback from the panelists closely reflects advice from other existing business sources, meaning that VC panels are a credible means of educating academic entrepreneurs and would-be entrepreneurs in the workings of a pitch, the creation of a business plan, and the process of fund raising.

## Conclusions

This paper has identified an inherent gap in business knowledge and training that may impede the translation of medical and psychological research into applied products, the commercialization of medical technologies, and the development of early stage health tech companies. We demonstrated that the gap reduces the chances of health care professionals engaging in medical start-ups and seeing their research insights implemented beyond the laboratory. Without business know-how, these professionals are less likely to successfully raise funds to support their companies and bring their ideas to fruition. The implications of this gap in knowledge go beyond the level of the individual academic entrepreneur and affect the entire health care industry. Medical and health care solutions developed in university settings can evolve into scalable intervention-based services and devices. Granted, there may be structural barriers to innovation and technology transfer, yet their focus would not necessarily be education. Hence, they were beyond the scope of this paper.

This paper discusses an additional solution: the inclusion of VC panels in medical conferences. These panels, which mirror a similar type of panel common in business conferences, have been organized at the Medicine 2.0 conference and have drawn considerable crowds and multiple submissions from companies, suggesting that all parties involved see potential gain in them and are willing to engage. The long-term effect of these panels can be evaluated by changes in the numbers of universities implementing existing solutions, in the generation of new solutions (mostly ones that overcome the locality issue), and in the ultimate creation of start-up companies in academia.

Similar to other solutions, VC panels are only a partial remedy to the lack of business knowledge of health care professionals. Rather than attempt to fix the problem in its entirety, VC panels can give both the companies presenting their products and the audience in attendance a chance to see how the business world functions. We regard the panels not only as a means of bridging the knowledge gap, but also as a way of sending a clear message to academicians and researchers: no matter how good your ideas are, you need to be able to understand how the business world works if you want to bring them to fruition. This can be an important teaching experience for health care professionals and researchers who are interested in developing products and services, and it is an experience that they are unlikely to receive anywhere else in the current medical educational system.

## Abbreviations

AMA: American Medical Association
CEO: chief executive officer
EET: entrepreneurship education and training
FDA: Food and Drug Administration
GCEC: Global Consortium of Entrepreneurship Centers
HIMSS: Health Information Management Systems Society
HIPAA: Health Insurance Portability and Accountability Act
MBA: Master of Business Administration
VC: venture capital
